# APP processing in Alzheimer's disease

**DOI:** 10.1186/1756-6606-4-3

**Published:** 2011-01-07

**Authors:** Yun-wu Zhang, Robert Thompson, Han Zhang, Huaxi Xu

**Affiliations:** 1 Institute for Biomedical Research, Xiamen University, 422 SiMingNanLu, Xiamen 361005, Fujian, PR China; 2 Neurodegenerative Disease Research Program, Sanford-Burnham Institute for Medical Research, 10901 North Torrey Pines Road, La Jolla 92037, CA, USA

## Abstract

An important pathological feature of Alzheimer's disease (AD) is the presence of extracellular senile plaques in the brain. Senile plaques are composed of aggregations of small peptides called β-amyloid (Aβ). Multiple lines of evidence demonstrate that overproduction/aggregation of Aβ in the brain is a primary cause of AD and inhibition of Aβ generation has become a hot topic in AD research. Aβ is generated from β-amyloid precursor protein (APP) through sequential cleavages first by β-secretase and then by γ-secretase complex. Alternatively, APP can be cleaved by α-secretase within the Aβ domain to release soluble APPα and preclude Aβ generation. Cleavage of APP by caspases may also contribute to AD pathologies. Therefore, understanding the metabolism/processing of APP is crucial for AD therapeutics. Here we review current knowledge of APP processing regulation as well as the patho/physiological functions of APP and its metabolites.

## Background

Alzheimer's disease (AD) is the most prevalent neurodegenerative disorder, afflicting 10% of the population over the age of 65 and 50% of the population over the age of 85. A small subset (<10%) of AD cases result from an inherited autosomal dominant gene mutation and have an early-onset (the fourth to sixth decade). The majority of these familial AD (FAD) mutations are in the genes encoding β-amyloid precursor protein (APP) and presenilins (PS1 and PS2) [1-3]. Significant efforts have gone into understanding the mechanisms underlying the genes tied to FAD as the clinicopathological features are indistinguishable from regular onset AD.

AD is characterized in patients by an inexorably progressing dementia. In vulnerable brain regions, such as the hippocampus and cortex, there is an accumulation of extracellular neuritic plaques and intracellular neurofibrillary tangles. The neurofibrillary tangles (NFTs) consist largely of hyperphosphorylated twisted filaments of the microtubule-associated protein tau [4,5]. Extracellular neuritic plaques are deposits of differently sized small peptides called β-amyloid (Aβ) that are derived via sequential proteolytic cleavages of the β-amyloid precursor protein (APP) [6].

### APP and Its Function

The *APP *gene is located on chromosome 21 in humans with three major isoforms arising from alternative splicing [3]. These are APP695, APP751 and APP770 (containing 695, 751, and 770 amino acids, respectively). APP751 and APP770 are expressed in most tissues and contain a 56 amino acid Kunitz Protease Inhibitor (KPI) domain within their extracellular regions. APP695 is predominantly expressed in neurons and lacks the KPI domain [7,8]. There are reports showing that the protein and mRNA levels of KPI-containing APP isoforms are elevated in AD brain and associated with increased Aβ deposition [9]; and prolonged activation of extrasynaptic NMDA receptor in neurons can shift APP expression from APP695 to KPI-containing APP isoforms, accompanied with increased production of Aβ [10]. These findings may suggest that a dysregulated splicing of *APP *RNA contributes to disease pathogenesis.

APP belongs to a protein family that includes APP-like protein 1 (APLP1) and 2 (APLP2) in mammals [11-13], all are type-I transmembrane proteins and are processed in a similar fashion. The Aβ domain is unique to the APP protein, though the family shares several other conserved domains such as the E1 and E2 domains in the extracellular sequence. Studies with *APP *knockout mice suggest some functional redundancy between these APP homologs that appears to be exerted by motifs other than Aβ. APP knockout mice are viable and fertile, showing a relatively subtle abnormal phenotype [14,15]. APLP1 and APLP2 knockout mice are also viable and fertile, though *APP/APLP2 *and *APLP1/APLP2 *double null mice and *APP/APLP1/APLP2 *triple null mice show early postnatal lethality [16-18]. Interestingly, the APP/APLP1 double null mice are viable [17], suggesting that *APLP2 *is crucial when either *APP *or *APLP1 *is absent.

**Figure 1:**
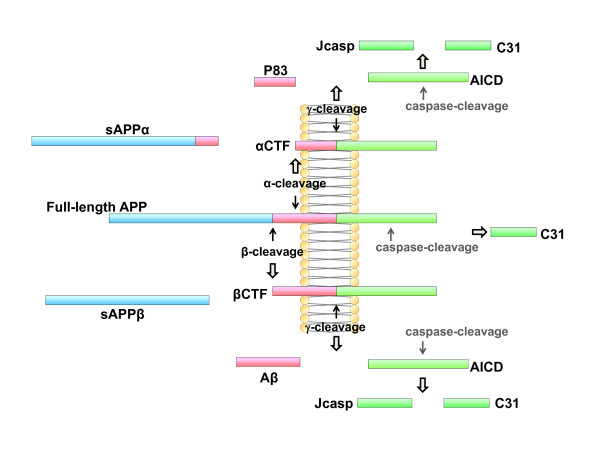
**Schematic diagram of APP processing (not drawn in proportion)**. It is not clear whether caspases cleave membrane-associated APP forms or released AICD.

Although APP has been the subject of much study since its identification, its physiological function remains largely undetermined. A role for APP has been suggested in neurite outgrowth and synaptogenesis, neuronal protein trafficking along the axon, transmembrane signal transduction, cell adhesion, calcium metabolism, etc, all requiring additional *in vivo *evidence (reviewed in [19]). APP is proteolyzed into various fragments (see Figure 1) during its intracellular trafficking and these APP metabolites mediate various and sometimes adverse functions. Therefore, the net effect of full-length APP on cellular activity may be a combination of its metabolites' functions, temporospatially depending on the proportion of levels of each APP metabolite. Here we list several possible functions of full-length APP *per se*.

The similarity in topology and proteolytic processing between APP and Notch suggest that APP may function as a membrane receptor like Notch. Indeed, several APP ligands have been identified, such as Aβ [20], F-spondin [21] and nectrin-1 [22]. However, while binding of APP by these ligands can affect APP processing, the exact downstream signaling events triggered by such binding remains to be clarified and a *bona fide *membrane receptor function for APP remains speculative.

There is evidence linking APP to cell adhesion. APP was found to colocalize with β1 intergrins in neural cells [23]. An X-ray analysis showed that the E2 domain of APP can form antiparallel dimers [24]. Indeed, further study in cell cultures demonstrated that APP can form homodimers and heterodimers in a trans-dimerization manner with other APP family members and that such dimerization promotes intercellular adhesion [25].

APP undergoes rapid anterograde transport in neurons. During its transport, APP was found to interact with kinesin-I and functions as a kinesin-I membrane receptor to mediate axonal transport of β-secretase (BACE1) and PS1 [26,27]. However, another study failed to verify the interaction between APP and kinesin-I and the co-transport of BACE1 and PS1 with APP [28]. We recently found that APP and its derived membrane-associated form, CTFs, can regulate cell surface delivery of PS1/γ-secretase but not BACE1 [29]. In addition, APP was found to be a major component of herpes simplex viral particles and likely mediates fast anterograde transport of these particles [30,31]. Another study showed that increased doses of APP markedly decreased retrograde transport of nerve growth factor and resulted in degeneration of forebrain cholinergic neurons in a mouse model of Down's Syndrome [32]. APP was also found to interact with high-affinity choline transporter (CHT) through the C-terminal domain and APP deficiency affected CHT endocytosis [33]. Overall, most studies suggest that APP plays some role in regulating protein trafficking.

### APP Processing

Full-length APP is a type I transmembrane protein. APP is synthesized in the endoplasmic reticulum (ER) and then transported through the Golgi apparatus to the trans-Golgi-network (TGN) where the highest concentration of APP is found in neurons at steady state [34-36]. Aβ is generated in the ER and Golgi/TGN [36]. From the TGN, APP can be transported in TGN-derived secretory vesicles to the cell surface where it is either cleaved by α-secretase to produce a soluble molecule, sAPPα [37], or re-internalized via an endosomal/lysosomal degradation pathway [38,39]. It has been proposed that Aβ can also be generated in the endosomal/lysosomal system [40,41]. While Aβ is neurotoxic, studies suggest that sAPPα is neuroprotective, making the subcellular distribution of APP an important factor in neurodegeneration [42-44]. Delineation of the mechanisms involved in APP trafficking are thus relevant and crucial to understanding the pathogenesis of AD.

#### α-secretase and α-processing

Cleavage of APP by α-secretase precludes Aβ generation as the cleavage site is within the Aβ domain (at the Lys16-Leu17 bond), and releases a large soluble ectodomain of APP called sAPPα. The generation of sAPPα is a constitutive event but can also be regulated by various reagents. Early studies suggested that α-secretase is a membrane-bound endoprotease which cleaves APP primarily at the plasma membrane [37]. Using proteinase inhibitor profiling, it was determined that α-secretase is a zinc metalloproteinase [45]. Several members of the ADAM (a disintegrin and metalloproteinase) family possess α-secretase-like activity and three of them have been suggested as the α-secretase: ADAM9, ADAM10, and ADAM17. Like APP, they are also type-I transmembrane proteins.

ADAM17 (also called tumor necrosis factor-α converting enzyme, TACE) can be proteolytically cleaved to release its extracellular domain as soluble TGF-α [46]. Manipulation of ADAM17 can alter α-cleavage of APP and Aβ generation, with regulated α-cleavage abolished in ADAM17-deficient cells, suggesting that ADAM17 is likely the α-secretase responsible for regulated APP cleavage [47]. Additionally, an ADAM17 inhibitor prevented regulated α-secretase activity in human neurons [48], whereas RNAi downregulation of ADAM10 had no effect on α-cleavage of APP [49]. Various other studies confirm that ADAM17 likely affects regulated, but not constitutive, α-cleavage in various cell lines [50].

Co-expression of ADAM9 with APP promoted sAPPα production upon phorbol ester treatment, suggesting that ADAM9 possesses α-secretase activity [51]. However, RNAi of ADAM9 had no effect on sAPPα generation [49], implying that ADAM9 is involved only in regulated α-cleavage.

Overexpression of ADAM10 increases α-cleavage, whereas a dominant-negative form of ADAM10 and RNAi of ADAM10 inhibit endogenous α-cleavage activity in several cell lines, including murine primary neurons [49,52,53]. Significantly, sAPPα generation was nearly abolished in the neurons of mice with neural ADAM10 conditionally knocked-out [54]. A dramatically reduced ADAM10 protein level in the platelets of sporadic AD patients was also found to correlate with the significantly decreased sAPPα levels found in their platlets and cerebrospinal fluid [55] and the reduced α-secretase activity in the temporal cortex homogenates of AD patients [56]. These studies strongly suggest that ADAM10 is the constitutive α-secretase that is active at the cell surface, though there may be some functional redundancy in α-cleavage among the ADAM family.

In contrast to Aβ, sAPPα has an important role in neuronal plasticity/survival and is protective against excitotoxicity [42,43]. sAPPα also regulates neural stem cell proliferation and is important for early CNS development [57,58]. We and others have also found that sAPPα can inhibit stress-induced CDK5 activation and participate in various neuroprotective reagent-mediated excitoprotection [44,59-61]. Interestingly, expression of sAPPα alone is able to rescue the abnormalities of APP deficient mice [62], implying that most of APP's physiological function is mediated by sAPPα.

#### β-secretase and β-processing

The first step in Aβ generation is cleavage of APP by the β-secretase. In 1999-2000, several groups concomitantly identified BACE1 (also called Asp2 or memapsin 2) as the major β-secretase [63-66]. BACE1, the most common name for the protease, is a membrane-bound aspartyl protease with a characteristic type I transmembrane domain near the C-terminus [63,64]. Overexpression or downregulation of BACE1 induces or inhibits cleavage of APP at the known β-site locations, Asp1 and Glu11, respectively. *In vitro *studies with synthetic APP peptides confirm cleavage by BACE1. These results provide convincing evidence that BACE1 is the β-secretase involved in APP metabolism [63-67]; and BACE1 activity is thought to be the rate-limiting factor in Aβ generation from APP.

A larger precursor, pro-BACE1, is modified by glycosylation, phosphorylation and cleaved by a furin-like endoprotease to produce mature BACE1 [68,69]. BACE1 requires an acidic environment for optimal activity and, as expected, overexpressed BACE1 in various pre-mitotic cell lines is mainly found in the early Golgi, late Golgi/early endosomes, and endosomes that provide an acidic environment. In addition, BACE1 can be found at the cell surface [64,70-72]. The mechanisms regulating BACE1 trafficking and activity have not been fully elucidated. Some studies found that BACE1 can interact with reticulon/Nogo proteins, whose increased expression can block BACE1 in the ER with a neutral pH environment and thus inhibit BACE1 activity in Aβ generation [73-75]. On the other hand, Golgi-localized γ-ear-containing ARF-binding (GGA) proteins have been found to interact with BACE1 and regulate its trafficking between the late Golgi and early endosomes; and depletion of GGA proteins increases the accumulation of BACE1 in acidic early endosomes for enhanced BACE1 stability and cleavage of APP [76-78].

The viability of BACE1 as a therapeutic target has been investigated by a number of studies. An early study suggested that BACE1 knockout mice do not produce detectable levels of Aβ and have no severe phenotypic abnormalities [79]. BACE1 deficiency in AD model mice have been shown to rescue cholinergic dysfunction, neuronal loss and memory deficits, correlating with a dramatic reduction in Aβ40/42 levels [79-81]. Several studies have found that BACE1 protein and activity levels are elevated in the regions of the brain affected by AD [82,83]. Together these results suggest BACE1 as a good therapeutic target for AD. However, more recent studies have found several phenotypic abnormalities in BACE1 KO mice. Dominguez et al. [84] observed a variable but significant number of BACE1 null mice died in the first weeks after birth. The BACE1 null mice that survive were smaller than their littermates, presented with hyperactive behavior, and had subtle electrophysiological alterations in the steady-state inactivation of their voltage-gated sodium channels. They also were affected by hypomyelination of peripheral nerves and had altered neurological behaviors such as reduced grip strength and elevated pain sensitivity, likely due to the deficiency of neuregulin processing in the absence of BACE1, as neuregulin 1 is another substrate of BACE1 [85,86]. Furthermore, additional BACE1 substrates have been identified, including the voltage-gated sodium channel (Nav1) β2 subunit, Golgi-localized membrane-bound α2,6-sialyltransferase, P-selectin glycoprotein ligand -1, etc. (reviewed in [87]). Therefore, BACE1 is likely not as safe a drug target as first assumed.

BACE2 is a homolog of BACE1 that maps to 21q22.3 [88], the region critical for Down's syndrome (DS). As DS also results in Aβ accumulation, the genes location suggests a link between BACE2 and APP processing. Indeed, BACE2 cleaves β-secretase substrates such as wild-type and Swedish mutant APP, similar to BACE1, in enzymatic *In vitro *assays [89]. However, BACE2 expression in neurons is substantially lower than BACE1 [90] and cellular BACE2 cleaves APP near the α-secretase site much more efficiently than at the β-secretase site [91]. These results suggest that BACE1 is the primary β-secretase but do not exclude a potential contribution of BACE2 towards AD pathogenesis. While BACE2 knockout mice are healthy overall, a deficiency of both BACE1 and BACE2 enhanced the BACE1 KO lethality phenotype, suggesting a slight functional redundancy [84].

In addition to BACE1 and BACE2, cathepsin B has been proposed as an additional β-secretase. Inhibition of cathepsin B has been found to reduce Aβ production both *in vivo *and *in vitro *[92,93]. However, whether cathepsin B really exerts physiological β-secretase activity requires further validation.

Upon β-cleavage, the ectodomain of APP is also released as soluble APPβ (sAPPβ). Although sAPPβ only differs from sAPPα by lacking the Aβ1-16 region at its carboxyl-terminus, sAPPβ was reported to function as a death receptor 6 ligand and mediate axonal pruning and neuronal cell death [94]. A recent report found that sAPPβ can rescue gene expression of transthyretin and Klotho, which is decreased in APP/APLP2 deficient mice, but cannot rescue the lethality and neuromuscular synapse defects of these mice, suggesting a gene expression regulation function for sAPPβ that is independent of developmental APP functions [95].

After α- and β-cleavage, the carboxyl terminal fragments (CTFs) of APP, known as αCTF and βCTF, respectively, remain membrane-associated and will be further cleaved by γ-secretase. Since these APP CTFs are intermediate products, their functions have been less characterized. However, overexpression of APP βCTF was found to be cytotoxic and cause neuronal degeneration, perhaps by perturbing APP signal transduction [96,97]. It is also possible that APP βCTF's cytotoxic effect is actually mediated by the end products of γ- and/or caspase-cleavage including APP intracellular domain (AICD), C31 and Jcasp which are cytotoxic (see below). We recently found that APP βCTF can regulate cell surface delivery of γ-sceretase, perhaps through the direct binding of enzyme-substrate [29]. It is possible that APP αCTF possesses a similar effect since it is also the substrate of γ-secretase.

#### γ-secretase and γ-processing

APP αCTF and βCTF are further cleaved by γ-secretase to generate p83 and Aβ, respectively. The p83 fragment is rapidly degraded and widely believed to possess no important function, if any. γ-secretase-mediated cleavage is unique in that the cleavage takes place within the transmembrane domain, though the exact site can vary. γ-cleavage can yield both Aβ40, the majority species, and Aβ42, the more amyloidogenic species, as well as release the intracellular domain of APP (AICD). Recent data has shown that PS/γ-secretase also mediates ζ-site cleavage (Aβ46) [98,99] and ε-site cleavage (Aβ49) [100,101], suggesting a sequential cleavage model where cleavage at the ε-site is followed by the ζ-site and γ-site.

Multiple lines of biochemical evidence have shown γ-secretase activity to reside in a high molecular weight complex consisting of at least four components: presenilin (PS, PS1 or PS2), Nicastrin, anterior pharynx-defective-1 (APH-1), and presenilin enhancer-2 (PEN-2) [102,103]. In mammals there are two presenilin homologs, PS1 and PS2 [1,2]. Mutations in these two genes, particularly PS1, are causative in the majority of familial AD (FAD) cases. PSs are multi-transmembrane proteins with an unclear number of transmembrane domains [104]. Nascent PSs undergo endroproteolytic cleavage with the resulting amino-terminal fragment (NTF) and carboxyl-terminal fragment (CTF) forming a functional PS heterodimer [105]. PSs possess two highly conserved aspartate residues indispensable for γ-secretase activity. The PS1 NTF/CTF heterodimers are bound by transition-state analogue γ-secretase inhibitors [102,106], suggesting that PSs are the crucial catalytic components of γ-secretase. This notion has recently been confirmed by *in vitro *assays [107]. Nicastrin, the first identified cofactor of PS, is a type I transmembrane glycoprotein that is considered the scaffolding protein within the γ-secretase complex. One study showed that the ectodomain of Nicastrin binds to APP and Notch and can recruit them into the γ-secretase complex, suggesting that Nicastrin may act as the γ-secretase receptor [108]. Another two components, APH1 and PEN2, were identified through genetic screening of *Caenorhabditis elegans *[109,110]. APH-1 interacts with Nicastrin to form a stable intermediate in an early assembly stage of the γ-secretase complex [102]. PEN-2 regulates PS endoproteolysis [107,108]. Each of these four γ-secretase components has been found necessary for the enzymatic activity of the complex with deficiency in any of them dramatically impairing γ-secretase activity. Coexpression of the four components in the yeast *Saccharomyces cerevisiae *has been found to be necessary and sufficient to reconstitute γ-secretase activity, which is not endogenous to yeast [111,112].

In addition to the four critical components, several other factors have been proposed as additional γ-secretase components. However, these factors play a modulatory role and are not essential for γ-secretase activity: CD147 is a transmembrane glycoprotein and interacts with all four essential γ-secretase components. Downregulation of CD147 increases Aβ production but its overexpression has no effect on Aβ generation [113]. TMP21/p23 binds to the γ-secretase complex and regulates γ-cleavage, but not ε-cleavage, through its transmembrane domain [114,115]. However, another study failed to confirm the binding of TMP23/p21 to γ-secretase, but rather suggested that TMP21/p23, which belongs to the p24 cargo family involved in vesicular trafficking regulation, influences APP trafficking and thus Aβ generation [116]. Recently, a novel γ-secretase activating protein (GSAP) was identified and GSAP was found to selectively increase Aβ production through interaction with both γ-secretase and the APP CTF substrate [117]. Additional validation and investigation of the role of these proteins in the γ-secretase complex is required.

Strong evidence suggests that the γ-secretase complex resides primarily in the ER, Golgi/TGN, endocytic and intermediate compartments--most of which (except the TGN) are not major subcellular localizations for APP [118,119]. In addition to cleaving APP CTFs, γ-secretase cleaves a series of functionally important transmembrane proteins, including Notch [120], cadherin [114], tyrosinase [121], ErbB4 [79], CD44 [70], etc.) (see review [122]). The cleavage of various substrates appears to be dependent on the subcellular compartment; APP is mainly cleaved in the TGN and early endosomal domains whereas Notch is primarily cleaved at the plasma membrane [34,36,123]. Thus a disturbance in the localization of the γ-secretase complex may play some role in abnormal Aβ generation and AD pathogenesis.

γ-cleavage can release the intracellular domains (ICDs) of the substrates. Notch intracellular domain (NICD) is well-known to translocate into the nucleus and regulate genes critical to development [124,125]. The other intracellular domains may be of comparable importance. For example, the ErbB4 ICD has been found to bind to astrocytic gene promoters to suppress their expression [126]. In a similar fashion, released AICD has been shown to possess transactivation activity and can regulate transcription of multiple genes including *APP*, *GSK-3β*, *KAI1*, *neprilysin*, *BACE1, p53, EGFR, and LRP1 *[127-132]. In addition, free AICD can induce apoptosis and may play a role in sensitizing neurons to toxic stimuli [133,134]. However, as the intracellular domain of APP, one important function of AICD is to facilitate the interaction of APP with various cytosolic factors that regulate APP's intracellular trafficking and/or signal transduction function. Interestingly, it seems that AICD-mediated APP interaction with different factors is controlled by the phosphorylation state of AICD [135].

#### Caspase processing

In addition to secretases, caspases (predominantly caspase-3) can directly cleave APP at position Asp664 (based on the APP695 sequence) within the cytoplasmic tail during apoptosis to release a fragment containing the last 31 amino acids of APP (called C31). Additional γ-cleavage further generates the fragment (called Jcasp) containing the region between γ- and caspase-cleavage sites [136-138]. Although original data found that caspase cleavage affects amyloidogenic processing of APP [137], further study suggests not [139]. However, during Aβ-induced neurotoxicity, activated caspases cleave APP to generate C31 and Jcasp, which are also neurotoxic, therefore initiating a detrimental cascade [140]. One possible mechanism for C31's toxicity is that C31 complexes with APP to recruit the interacting partners that initiate the signals related to cellular toxicity [136]. Compared to C31, Jcasp appears to play a minor role in cytotoxicity [136]. Importantly, caspase cleavage of APP seems to be crucial for Aβ-mediated neurotoxicity, as an APP mutation at position Asp664 to inhibit the caspase-cleavage in transgenic mice negated the synapse, electrophysiology, and behavioral abnormalities, even though Aβ plaques were still abundant in the brain [141].

### Aβ Function

The neurotoxic effect of Aβ has been well-established and will not be specifically emphasized here. Multiple lines of evidence demonstrate that overproduction of Aβ results in a neurodegenerative cascade leading to synaptic dysfunction, formation of intraneuronal fibrillary tangles and eventually neuron loss in affected areas of the brain [6,142]. There are two main toxic species, Aβ40 and Aβ42, with Aβ42 more hydrophobic and more prone to fibril formation while only making up about 10% of the Aβ peptide produced [143]. Studies done on familial AD (FAD) mutations consistently show increases in the ratio of Aβ42/40 [105,144], suggesting that elevated levels of Aβ42 relative to Aβ40 is critical for AD pathogenesis, probably by providing the core for Aβ assembly into oligomers, fibrils and amyloidogenic plaques [145,146].

Although the majority of Aβ is secreted out of the cell, Aβ can be generated in several subcellular compartments within the cell, such as the ER, Golgi/TGN, and endosome/lysosome. In addition, extracellular Aβ can be internalized by the cell for degradation. The intracellular existence of Aβ implies that Aβ may accumulate within neurons and contribute to disease pathogenesis. Confirming this, intraneuronal Aβ immunoreactivity has been found in the hippocampal and entorhinal cortical regions which are prone to early AD pathology in patients with mild cognitive impairment (MCI) [147]. In Down Syndrome (DS) patients, the accumulation of intracellular Aβ precedes extracellular plaque formation [148] and the level of intraneuronal Aβ decreases as the extracellular Aβ plaques accumulate [149]. Studies with transgenic mouse models consistently confirm these results, revealing intracellular Aβ accumulation as an early event in the neuropathological phenotype with decreasing intraneuronal levels of Aβ as extracellular plaques build up [150-152]. Intraneuronal Aβ can also impair amygdala-dependent emotional responses by affecting the ERK/MAPK signaling pathway [153]. Inhibition of dynamin-mediated but not clathrin-mediated Aβ internalization was also found to reduce Aβ-induced neurotoxicity [154]. One recent study suggests that internalized Aβ can aggregate within the cell and disrupt the vesicular membrane, thus contributing to its pathological effect [155].

Aβ was originally regarded as an abnormal and toxic species restricted to the brains of aged or demented humans. The discovery of soluble Aβ species in the bodily fluids of various species [156] and in the conditioned medium of cultured cells [157] has refuted this concept and implied a physiological function for Aβ. Although excessive Aβ causes synaptic dysfunction and synapse loss [142], low levels of Aβ increase hippocampal long-term potentiation and enhances memory, indicating a novel positive, modulatory role on neurotransmission and memory [158,159]. Picomolar levels of Aβ can also rescue neuronal cell death induced by inhibition of Aβ generation (by exposure to inhibitors of β- or γ-scretases) [160], possibly through regulating the potassium ion channel expression, hence affecting neuronal excitability [161]. One study using a transgenic *Caenorpabditis elegans *model found that intracellular Aβ aggregation in muscle cells may trap excess free copper to reduce copper-mediated cytotoxic effects [162]. However, whether Aβ can form intracellular aggregates in human peripheral cells to exert a physiologically protective function remains to be determined.

### Regulation of APP Processing at the Trafficking Level

Alterations in APP intracellular trafficking and localization directly impact Aβ production as APP is processed by two mutually exclusive pathways. The available evidence has shown that intracellular trafficking of APP is regulated by a number of factors.

#### Trafficking factors

Intracellular trafficking of proteins requires the involvement of a series of cytosolic factors. An increasing number of proteins that interact with APP or act as trafficking factors are being implicated in the regulation of Aβ generation and APP trafficking. For example, the APP C-terminus has been found to interact with all three mint (X11) family members (mint1, mint2, and mint3) involved in trafficking regulation [163-165]. APP interaction with mint proteins has been shown to affect APP processing by stabilizing cellular APP, altering both sAPPα and Aβ generation and secretion [166]. Rab6, a member of the GTP-binding protein family of membrane trafficking regulators, is implicated in protein transport along biosynthetic and endocytic pathways and has also been found to affect APP processing. Moreover, internalization of APP from the cell surface for endosomal/lysosomal degradation can be mediated by clathrin. Clathrin-modulated endocytosis is tightly controlled, requiring the participation of AP-2, dynamin I, and many other factors [167-169]. When the endocytic pathway is inhibited by overexpression of a dominant-negative form of dynamin I, APP processing is also affected [170,171]. It is conceivable that other important vesicular transport factors may also affect APP processing through regulation of general protein trafficking.

In addition to general trafficking modulators, several other proteins have been found to regulate APP trafficking in a more specific manner, possibly through their direct binding to APP. PS1 is the catalytic component of the γ-secretase complex but has also been demonstrated to regulate the intracellular trafficking of several membrane proteins, including the other γ-secretase components (nicastrin, APH-1 and PEN-2), TrkB, and ICAM-5/telecephalin [122]. We and others have shown that PS1 can also regulate the intracellular trafficking of APP. Expression of a loss of function PS1 variant, or the absence of PS1, results in increased budding/generation of vesicles from both the ER and TGN containing APP along with a concomitant increase in complex glycosylation and APP localization at the cell surface. In contrast, the FAD-linked PS1 mutant variants significantly reduce budding from the ER and TGN and result in decreased delivery of APP to the cell surface [172]. These results suggest the possibility that FAD-linked PS1 variants increase Aβ production by decreasing intracellular transport of APP, prolonging the availability of APP for cleavage by β- and γ-secretases within the TGN. PS1 may regulate protein trafficking through its interaction with several cytosolic factors involved in the regulation of vesicular transport such as Rab11, Rab6 and Rab GDI [173-175]. We have also found that PS1 interacts with phospholipase D1 (PLD1), a phospholipid-modifying enzyme regulating membrane trafficking events. This PS1-PLD1 interaction recruits PLD1 to the Golgi/TGN and thus potentially alters APP trafficking as PLD1 overexpression promotes budding of vesicles from the TGN containing APP and increases cell surface levels of APP [176,177].

SorLA/LR11 is a type I membrane protein expressed in neurons and reduced in the brains of AD patients [178,179]. Although the function of SorLA/LR11 is not known, its homology with sorting receptors that are involved with transport between the plasma membrane, endosomes and the Golgi suggests a protein trafficking function [180,181]. Recently it was found that SorLA/LR11 overexpression redistributed APP to the Golgi, decreasing Aβ generation, while SorLA/LR11 knockout mice have increased levels of Aβ, as found in AD patients [182]. Additionally, some inherited variants of the *SorLA/LR11 *gene were found to associate with late-onset AD [183].

Low-density lipoprotein receptor-related protein (LRP) is a SorLA/LR11-related protein that binds to APP through Fe65, a cytoplasmic adaptor protein [184]. LRP has been shown to bind, directly or indirectly, with Aβ to mediate its clearance [185,186]. Antagonizing the extracellular interaction between cell-surface APP and LRP increased the level of cell surface APP while decreasing Aβ generation [187]. Using an AD mouse model, expression of a functional LRP minireceptor in neurons resulted in increased memory deficits and higher Aβ levels in the aged mice [188]. An LRP-related protein 1B (LRP1B) has a similar effect, binding APP at the plasma membrane, preventing APP internalization, and leading to decreased Aβ generation and increased sAPPα secretion [189].

#### Signal transduction

Epidemiological evidence suggests that post-menopausal women receiving replacement therapy of the sex hormone estrogen have a reduced risk and delayed onset of AD while elderly women with reduced levels of circulating estrogen have an increased incidence of AD [190-192]. The primary mechanism of estrogen's protection against AD development is still unclear. Several potential mechanisms have been proposed: (1) estrogen may act on interlukin 6 to antagonize inflammation [193]; (2) the phenolic structure of estrogen may contribute to its antioxidant effect in cells [194]; (3) estrogen may reduce the level of appolipoprotein E (ApoE), with the isoform ApoE4 being a strong risk factor for AD development [195]; and (4) gonadal steroids may reduce the protein level of PS1 and thus γ-secretase activity [196].

We have found that estrogen may reduce Aβ levels by stimulating the α-secretase pathway and thereby inhibit Aβ generation. Estrogen can stimulate the formation of APP-containing vesicles from the TGN in cell-free systems derived from both neuroblastomas and primary neurons [197-199]. Interestingly, the stimulation of sAPPα secretion by estrogen can be blocked by a PKC inhibitor, suggesting the involvement of a PKC-dependent pathway [200]. Indeed, phorbol ester's effect on sAPPα secretion and Aβ generation though activation of protein kinase C (PKC) has been known for a long time [201-203]. PKC stimulates sAPPα secretion, reducing Aβ levels, even when the phosphorylation sites on APP are mutated or the entire cytoplasmic domain is deleted [204]. While PKC can directly phosphorylate APP Ser655 [205], it appears to affect APP metabolism by phosphorylating a different target. One potential target is a TGN phosphoprotein, resulting in transport of APP from the TGN to the cell surface. Our studies have shown that PKC increases the formation of APP-containing secretory vesicles from the TGN in a cell-free system [206]. In support of this, protein kinase A (PKA) has similar effects on reducing Aβ generation and stimulating the budding of APP-containing vesicles from the TGN [207]. The effects of PKC and PKA are additive, suggesting that while they both appear to act through stimulating vesicle formation from the TGN, the regulatory mechanisms involved are independent [207]. Additionally, estrogen has been found to facilitate binding of Rab11 to the TGN membrane and a dominant negative Rab11 mutant abolishes the estrogen-regulated change in APP trafficking, leading to increased Aβ formation [197].

The sex hormone testosterone decreases with age in older men and postmenopausal women. Animal model studies of testosterone treatments show neuroprotective and neuroexcitatory benefits along with improved cognitive performance [208]. Some studies have suggested that testosterone exerts its beneficial effect through an aromatase-mediated conversion into estrogen [209,210]. However, a recent study blocking the conversion of testosterone to estrogen found an estrogen-independent improvement in cognitive function and lowering of plaque formation along with a decrease in BACE1 mRNA, protein level, and activity [211]. In addition, testosterone may also reduce the protein level of PS1 [196].

## Conclusion

The overproduction and accumulation of Aβ in the brain are key pathogenic events in AD progression. In addition to Aβ, APP can be proteolyzed by different secretases and caspases. In this review we have discussed APP processing regulation and the physio/pathological functions of various APP metabolites. Further elucidation of APP metabolism will be important for identifying new potential therapies to reduce Aβ accumulation and combat AD.
